# Polyacrylic acid, but not polyethylene glycol, induces metabolic reprogramming linked to pulmonary fibrosis in rats

**DOI:** 10.1038/s41598-025-33213-1

**Published:** 2025-12-18

**Authors:** Taisuke Tomonaga, Hiroto Izumi, Chinatsu Nishida, Kazuma Sato, Yuiko Nakamura, Shinya Morimoto, Toshiki Morimoto, Yasuyuki Higashi, Hidenori Higashi, Midori Iida, Takuma Kojima, Kazuo Sakurai, Akihiro Moriyama, Jun-ichi Takeshita, Kazuhiro Yatera, Yasuo Morimoto

**Affiliations:** 1 Department of Occupational Pneumology, Institute of Industrial Ecological Sciences, University of Occupational and Environmental Health, 1-1 Iseigaoka, Yahata-nishi-ku, Kitakyushu, 807-8555 Fukuoka Japan; 2 Department of Environmental Health Engineering, Institute of Industrial Ecological Sciences, University of Occupational and Environmental Health, Japan. 1-1 Iseigaoka, Yahata-nishi-ku, Kitakyushu, 807-8555 Fukuoka Japan; 3 Department of Respiratory Medicine, University of Occupational and Environmental Health, 1-1 Iseigaoka, Yahata-nishi-ku, Kitakyushu, 807-8555 Fukuoka Japan; 4 Department of Physics and Information Technology, Kyushu Institute of Technology, 680-4 Kawazu, Iizuka-shi, Fukuoka, 820-8502 Japan; 5 Department of Chemistry and Biochemistry, The University of Kitakyushu, 1-1, Hibikino, Wakamatsu-ku, Kitakyushu, 808-0135 Fukuoka Japan; 6 Research Institute of Science for Safety and Sustainability, National Institute of Advanced Industrial Science and Technology (AIST), 16-1 Onogawa, Tsukuba, Tsukuba, 305-8569 Ibaraki Japan; 7 Institute of Industrial Ecological Sciences, University of Occupational and Environmental Health, Iseigaoka, Yahata-nishi-ku, Kitakyushu, 807-8555 Fukuoka Japan

**Keywords:** Polyacrylic acid, Polyethylene glycol, Intratracheal instillation, Metabolomics, Fibrosis, Rat, Biochemistry, Biomarkers, Diseases, Medical research

## Abstract

Polyacrylic acids are widely used in industrial and consumer products, yet increasing evidence indicates that they can induce severe lung injury and rapidly progressing fibrosis. The underlying mechanisms remain unclear. In this study, we investigated the pulmonary toxicity of polyacrylic acid (A45) in comparison with polyethylene glycol (PEG, negative control) in a rat intratracheal instillation model. Lung inflammation and fibrosis were assessed up to 6 months. To explore systemic responses to pulmonary injury, serum metabolomics was performed using capillary electrophoresis time-of-flight mass spectrometry (CE-TOFMS). A45 exposure caused marked neutrophil infiltration, elevated inflammatory markers, and persistent fibrotic remodeling, while PEG induced no significant pulmonary changes. Metabolomic profiling detected 249 metabolites, of which 182 passed quality filtering. A45 significantly altered amino acid, energy, and redox pathways, whereas PEG showed minimal changes. Differential metabolites included valine, leucine, alanine, glutamine, malate, carnitine, cysteine, and methionine sulfoxide. Pathway analysis revealed enrichment of amino-acid/collagen-related and energy-demanding pathways. These findings demonstrate that polyacrylic acid induces systemic metabolic reprogramming linked to oxidative stress, mitochondrial dysfunction, and fibrosis, highlighting serum metabolomics as a valuable tool for mechanistic toxicology and biomarker discovery.

**Supplementary Information:**

The online version contains supplementary material available at 10.1038/s41598-025-33213-1.

## Introduction

Organic chemicals comprise nearly all carbon-containing compounds, excluding simple inorganic forms such as carbon oxides. Traditionally, it was considered that they cause allergic airway inflammation or hypersensitivity pneumonitis due to immune-mediated reactions, but not pneumoconiosis^1^. Recent evidence, however, has revealed that certain organic chemicals can induce persistent inflammation and pulmonary fibrosis. For example, polyhexamethylene guanidine phosphate (PHMG-p) and ethoxyethylguanidine chloride (PGH), ingredients in humidifier disinfectants in South Korea, were identified as causative agents of fatal pulmonary fibrosis^2,3^. In the United States, e-cigarette use has been associated with acute respiratory failure^4,5^. In Japan, workers exposed to water-absorbent cross-linked polymers, including polyacrylic acids widely used in consumer products such as diapers, shampoos, cosmetics, and food additives, developed airway inflammation and progressive pulmonary fibrosis^6–9^. Notably, fibrosis appeared within two years after exposure and progressed more rapidly than lung disorders caused by asbestos or crystalline silica^6–9^.

Animal studies have reinforced these observations. Both intratracheal instillation and inhalation exposure to polyacrylic acids induced lung inflammation and fibrosis in rodents^8,10,11^. Histopathological examinations demonstrated rapid fibrotic progression, consistent with findings from human cases. These data strongly indicate that polyacrylic acids are fibrogenic in vivo, yet the molecular mechanisms driving this pathology remain incompletely understood. In particular, how polymer exposure perturbs cellular metabolism and promotes the transition from inflammation to fibrosis is a critical, unanswered question.

Metabolism represents the most proximal phenotype of biological activity, bridging gene and protein regulation with functional outcomes^12^. Metabolomic analysis can therefore provide unique insights into the pathophysiology of lung injury and fibrosis^13,14^. It has also emerged as a powerful tool for biomarker discovery and disease diagnosis^15–18^. Among analytical platforms, capillary electrophoresis time-of-flight mass spectrometry (CE-TOFMS) is especially suitable for profiling small ionic and water-soluble molecules such as sugars, amino acids, and nucleotides^19–21^.

Here, we aimed to elucidate the mechanisms of polyacrylic acid–induced lung injury and fibrosis by identifying systemic metabolic alterations. Using CE-TOFMS–based metabolomics of rat serum after intratracheal instillation, we compared polyacrylic acid (A45, a fibrogenic polymer) with polyethylene glycol (PEG, a biocompatible negative control). Our goal was to characterize the metabolic pathways perturbed by polyacrylic acid exposure and to provide mechanistic insight into the development of lung inflammation and fibrosis.

## Methods

### Preparation of polymer samples

Polyacrylic acid (A45) (≤ 0.1% crosslinker, Weight average molecular weight (Mw) 7.65 × 10^5^ g/mol; Sigma-Aldrich, St. Louis, MO, USA; catalog no. 181285) and polyethylene glycol (PEG) (Mw 2.02 × 10^3^ g/mol; Wako Pure Chemical Corporation, Osaka, Japan; catalog no. 168–11285) were used in this study. Both compounds were supplied as white powders. Each polymer was suspended in distilled water at a final concentration of 2.5 mg/mL and stirred slowly for 40 min using a magnetic stirrer (Mag-Mixer MF820, Yamato Scientific Co., Ltd., Tokyo, Japan). The suspensions were freshly prepared before administration.

### Animals and ethical approval

Male Fischer 344 rats (8 weeks old, Charles River Laboratories Japan, Kanagawa, Japan) were acclimated for 4 weeks in the Laboratory Animal Research Center of the University of Occupational and Environmental Health, Japan, with free access to a standard commercial diet (manufacturer, catalog no.) and tap water under controlled environmental conditions (22 ± 2 °C, 55 ± 10% humidity, 12 h light/dark cycle). At the time of the experiments, 12-week-old male rats (body weight: 255.6 ± 13.3 g) were used for intratracheal instillation.

All animal procedures were approved by the Animal Care and Use Committee of the University of Occupational and Environmental Health, Japan (Approval No. AE17-009, AE19-008), and were conducted in accordance with the Japanese Guide for the Care and Use of Laboratory Animals and the ARRIVE guidelines (https://arriveguidelines.org).

### Intratracheal instillation procedure

Rats (12 weeks old) were anesthetized with sevoflurane (2–3% in oxygen; Viatris Inc., Canonsburg, PA, USA) and subjected to a single intratracheal instillation. Doses of 0.2 mg (0.8 mg/kg body weight) and 1.0 mg (4.0 mg/kg body weight) of A45 or PEG suspended in 0.4 mL distilled water were administered. These maximum doses were determined to avoid pulmonary overload, which can induce not only toxicity attributable to the chemicals themselves but also nonspecific toxicity resulting from impaired clearance in the lung. This dose setting was based on our previous studies^10,22,23^. A control group received 0.4 mL of distilled water alone.

The procedure was performed as previously described^22,23^. Briefly, the larynx was visualized using a laryngoscope (MAC1, Rudolf Riester GmbH, Jungingen, Germany), and an animal feeding needle (KN-348, Natsume Seisakusho Co., Ltd., Tokyo, Japan) was inserted into the trachea for manual instillation of the suspension. To prevent sample retention, approximately 3 mL of air was gently introduced twice via the feeding needle. No alveolar damage was observed under these conditions in our previous experiments^22,23^. After instillation, the rats were allowed to recover spontaneously and were monitored periodically.

To minimize the risk of cross-contamination during the intratracheal instillation procedures, the tracheal cannula was thoroughly washed with ultrapure water after each instillation. For each exposure group, the cannula was replaced with a newly prepared, sterilized cannula. All plastic instruments used for bronchoalveolar lavage fluid (BALF) collection and subsequent analyses of cellular and protein components were autoclave-sterilized prior to use and were handled as disposable materials. These procedures were implemented routinely to ensure that contamination was minimized throughout the experimental process.

### Animal groups and sample collection

Each exposure and control group consisted of five rats, consistent with our previous studies^22,23^. Rats were sacrificed at 3 days, 1 week, 1 month, 3 months, and 6 months after intratracheal instillation. Body weight, lung weight, and BALF volume were recorded, and BALF and lung tissue samples were collected at necropsy.

Under deep isoflurane anesthesia (3–4% in oxygen; Viatris Inc., Canonsburg, PA, USA), body weight of each rat was measured. After confirming a surgical level of anesthesia, the thoracic cavity was opened, and blood was collected from the heart. Euthanasia was confirmed by cessation of heartbeat and respiration. The lungs were then perfused with saline and excised using standard dissection procedures. With the left main bronchus clamped, the right lung was lavaged twice with saline at a pressure of 20 cm H₂O, yielding BALF for biochemical analysis. After lavage, the right and left lungs were separated: the right lung was used for heme oxygenase-1 (HO-1) measurement, and the left lung was inflated and fixed with 10% formaldehyde at 25 cm H₂O for histopathological evaluation.

### BALF cytology and inflammatory marker measurements

BALF was centrifuged at 400 × g for 15 min at 4 °C. The supernatant was collected for measurement of total protein, lactate dehydrogenase (LDH), and cytokines, and the cell pellet was washed with polymorphonuclear leukocyte (PMN) buffer (137.9 mM NaCl, 2.7 mM KCl, 8.2 mM Na₂HPO₄, 1.5 mM KH₂PO₄, and 5.6 mM glucose), recentrifuged under the same conditions, and resuspended in 1 mL PMN buffer.

Total cell counts were determined using an automated cell counter (ADAM-MC, AR Brown, Tokyo, Japan). Cytospin preparations were made on glass slides, fixed, and stained with Diff-Quik (Sysmex, Kobe, Japan). Neutrophils and alveolar macrophages were quantified by microscopic observation.

LDH activity in the BALF supernatants was determined with a Cytotoxicity Detection KitPLUS (Roche Diagnostics, Mannheim, Germany), using a standard curve generated from recombinant rabbit muscle LDH (Oriental Yeast, Tokyo, Japan). Protein concentrations were measured with the Pierce™ 660 nm Protein Assay (Thermo Scientific, Rockford, IL, USA) according to the manufacturer’s instructions.

### BALF chemokine and lung HO-1 measurements

Concentration of cytokine-induced neutrophil chemoattractants-1 (CINC-1) in the BALF was measured using ELISA kits (#RCN100, R&D Systems, Minneapolis, MN, USA) according to the manufacturer’s instructions.

For HO-1 measurements, the third lobes of the right lungs were homogenized in T-PER tissue protein extraction reagent (Thermo Scientific, Rockford, IL, USA) supplemented with protease inhibitor cocktails (P8340, Sigma-Aldrich, St. Louis, MO, USA; cOmplete Mini, Roche Diagnostics, Mannheim, Germany). Homogenates were centrifuged at 20,400 × g for 10 min at 4 °C, and protein concentrations of the supernatants were determined using the Pierce™ 660 nm Protein Assay (Thermo Scientific, Rockford, IL, USA) with bovine serum albumin as a standard. HO-1 concentrations were quantified with an ELISA kit (ADI-EKS-810 A, Enzo Life Sciences, Farmingdale, NY, USA) and normalized to total protein concentration.

### Histopathological evaluation

Formalin-fixed lung tissues were embedded in paraffin, sectioned at 4 μm, and stained with hematoxylin and eosin (HE) and Masson’s trichrome (MT). Pulmonary fibrosis was assessed using the modified Ashcroft scoring system^21^, as described previously^22,23^. Histopathological findings were graded on a scale of 0 to 8, and mean ± standard deviation values were calculated for each group. Scoring was performed by two independent observers blinded to the experimental groups.

### Metabolite extraction from serum

Metabolites were extracted from 50 µL of serum by adding 200 µL of methanol containing internal standards (H3304-1002; Human Metabolome Technologies, Inc. (HMT, Tsuruoka, Yamagata, Japan) at 0 °C to inhibit enzymatic activity. After thorough mixing, 150 µL of Milli-Q water was added, and 300 µL of the mixture was filtered by centrifugation through a 5-kDa cutoff filter (ULTRAFREE MC PLHCC; HMT) at 9,100 × g for 30 min at 4 °C to remove macromolecules. The filtrate was evaporated to dryness under vacuum and reconstituted in 50 µL of Milli-Q water for subsequent CE-TOFMS analysis at HMT. To reduce potential interference from high–molecular-weight contaminants, including laboratory-derived PEG, the extraction protocol used only methanol, ultrapure water, and defined internal standards, without any PEG-containing reagents, and the 5-kDa ultrafiltration step largely removed macromolecules such as typical laboratory PEG polymers in the kDa range. The CE-TOFMS Basic Scan platform employed in this study is targeted to small ionic primary metabolites (e.g., amino acids, organic acids, and sugars), and PEG polymers are not included in the annotated metabolite panel; all serum samples from all experimental groups were processed in an identical manner using the same types of tubes and pipette tips to avoid systematic biases.

### Metabolomic profiling

Metabolomic analysis was performed using capillary electrophoresis time-of-flight mass spectrometry (CE-TOFMS; 6230, Agilent Technologies) at Human Metabolome Technologies, Inc. (Tsuruoka, Japan), based on the methods described previously^21,24^. The systems were controlled by Agilent MassHunter Workstation Data Acquisition (Agilent Technologies) and connected by a fused silica capillary (50 μm i.d. × 80 cm total length) with commercial electrophoresis buffer (H3301-1001 and I3302-1023 for cation and anion analyses, respectively, HMT) as the electrolyte. The spectrometer was scanned from m/z 50 to 1,000 and peaks were extracted using MasterHands, automatic integration software (Keio University, Tsuruoka, Yamagata, Japan) in order to obtain peak information including m/z, peak area, and migration time^25,26^. Signal peaks corresponding to isotopomers, adduct ions, and other product ions of known metabolites were excluded, and the remaining peaks were annotated according to HMT’s metabolite database based on their m/z values and migration times. Areas of the annotated peaks were then normalized to internal standards and sample amount in order to obtain relative levels of each metabolite. L-methionine sulfone and D-camphor-10-sulfonic acid were used as internal standards for cationic and anionic metabolites, respectively. These intensity tables were obtained, and missing values were imputed as zero. Intensities were log2-transformed and scaled after batch correction using the ComBat algorithm. After removing constant features, a final matrix of 182 metabolites across 20 samples (PEG controls, PEG-treated, A45 controls, and A45-treated) was used for statistical analyses.

Importantly, the PEG control (PEG Ctrl) and A45 control (A45 Ctrl) groups originated from separate experimental batches. To evaluate potential batch effects, PCA and PERMANOVA were first applied to compare the two control groups directly. Although no significant differences were observed (PERMANOVA, *p* = 0.095, trend-level), the two controls were conservatively treated as independent in all downstream analyses in all downstream analyses to ensure that treatment effects were interpreted relative to their respective matched control.

### Data visualization

Volcano plots were generated for each comparison (A45 vs. A45 Ctrl, PEG vs. PEG Ctrl, A45 vs. PEG). Sankey (alluvial) diagrams were constructed using ggalluvial to visualize metabolite–pathway–phenotype relationships. Metabolite colors were assigned as red for upregulated and blue for downregulated metabolites. The widths of the flows represent visual layout adjustments and do not encode quantitative information. Heatmaps of significant metabolites (FDR < 0.05, no fold-change cutoff) were also generated for each comparison to visualize clustering patterns among the samples. Violin plots with overlaid dots and boxplots were used to show biomarker distributions.

### Pathway annotation and categorization

Metabolite annotation was performed using PubChem CID, and HMDB ID. KEGG compound identifiers were mapped via KEGGREST. Pathway associations were retrieved from KEGG rat (rno) pathway databases^27,28^, with human/mammalian maps substituted when rat annotations were not available. Non-mammalian pathways (e.g., plant or microbial-specific) were grouped as “Other pathway” to avoid spurious associations. Long pathway names were shortened for clarity (e.g., biosynthesis → biosyn., metabolism → metab.). To facilitate biological interpretation, each metabolite/pathway was further categorized into phenotypic groups: amino acid metabolism, energy metabolism, inflammation-related, fibrosis-related, oxidative stress–related, or other.

### Statistical analysis

Dunnett tests using IBM SPSS Statistics version 25 (https://www.ibm.com/jp-ja/products/spss-statistics) (IBM Corporation, Chicago, IL, USA) were appropriately used to detect differences between the rats exposed to A45 and PEG samples and each control group, and p-values < 0.05 were determined to be statistically significant.

Principal component analysis (PCA) was performed to assess global separation of the treatment and control groups. Because two independent control groups were included (PEG Ctrl and A45 Ctrl), the treatment effects were always tested against the corresponding matched control. Differential metabolite abundance (A45 vs. A45 Ctrl, PEG vs. PEG Ctrl, and A45 vs. PEG; *n* = 5 per group) was analyzed using the limma linear model with empirical Bayes moderation. False discovery rate (FDR) was controlled using the Benjamini–Hochberg procedure, with significance defined as FDR-adjusted *p* < 0.05. All analyses, except for the Dunnett tests, were performed in R version 4.5.1 using the limma, ggplot2, pheatmap, and ggalluvial packages.

## Results

### Characterization of polyethylene glycol and polyacrylic acid

The fundamental characteristics of the PEG and A45 used in this study are summarized in Table 1. The A45 was the same that was used in our previous study as a cross-linked polyacrylic acids^22^. Gel permeation chromatography (GPC) analysis (Prominence 501 system coupled with a Dawn-Heleos-Ⅱ, Wyatt Technology Europe GmbH, Dernbach, Germany) using GF-7MHQ (Showa Denko K.K., Tokyo, Japan) with 0.1 M carbonate–bicarbonate buffer as the eluent revealed that the weight-average molecular weights (Mw) of the PEG and A45 were 2 × 10³ and 7.6 × 10⁵, respectively^29,30^. The PEG formed a viscous paste when dispersed in solvent, and its radius of gyration (Rg) could not be determined due to its low molecular weight.

**Table 1:** Physiochemical characterization of polyethylene glycol and polyacrylic acid.

Physiochemical characterization	Polyethylene glycol (PEG)	Crosslinked Polyacrylic acid (A45)
Structural formula		
Weight average molecular weight (Mw)	2.02 × 10^3^ g/mol	7.65 × 10^5^ g/mol
Number average molecular weight (Mn)	1.59 × 10^3^ g/mol	4.14 × 10^5^ g/mol
Poly dispersity index (PDI)	1.27	1.85
Degree of crosslinking	None	~ 0.1%
Radius of gyration (Rg)	-	68.7 nm

### Body weight changes after intratracheal instillation

**Fig. 1:**
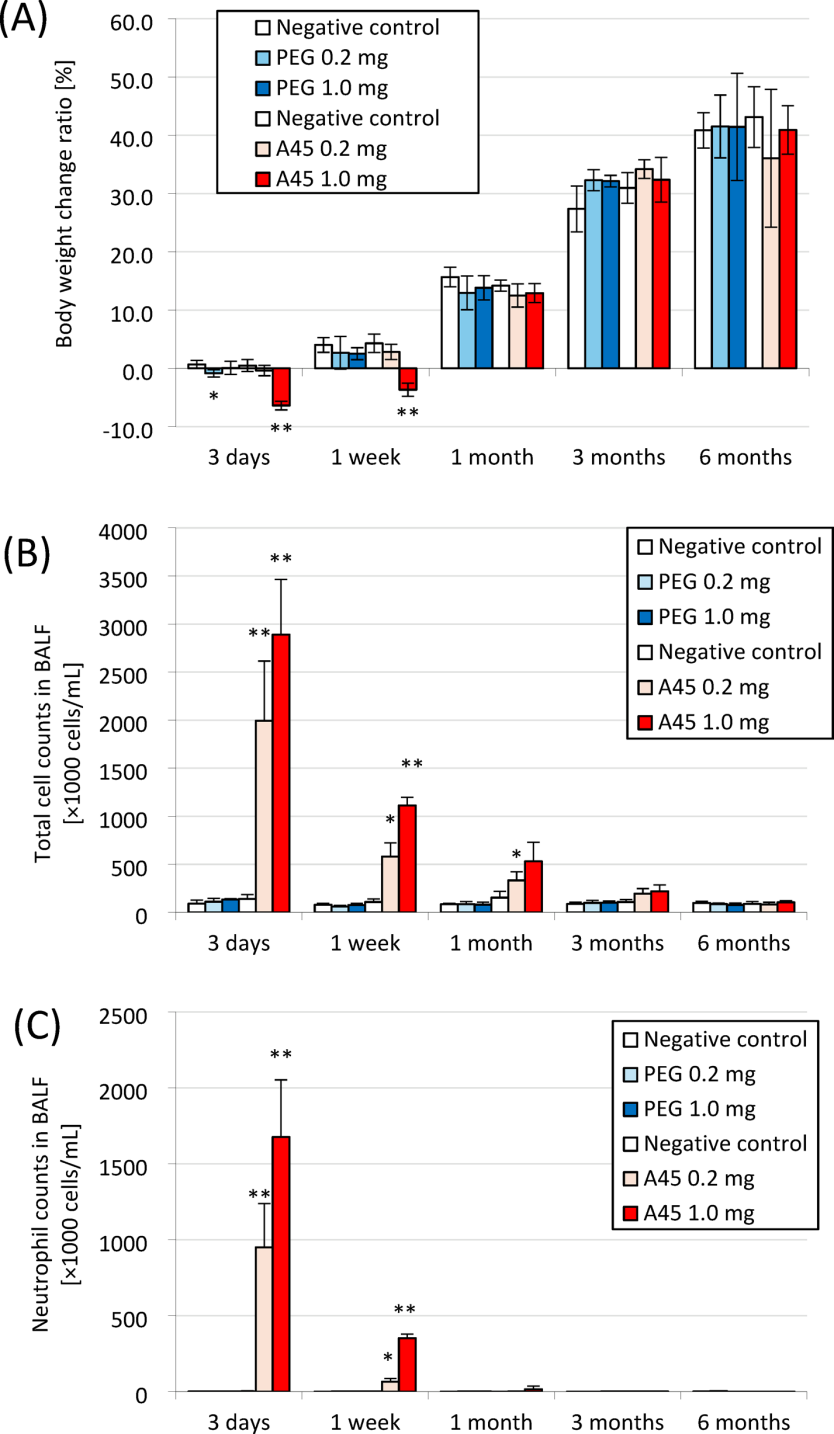

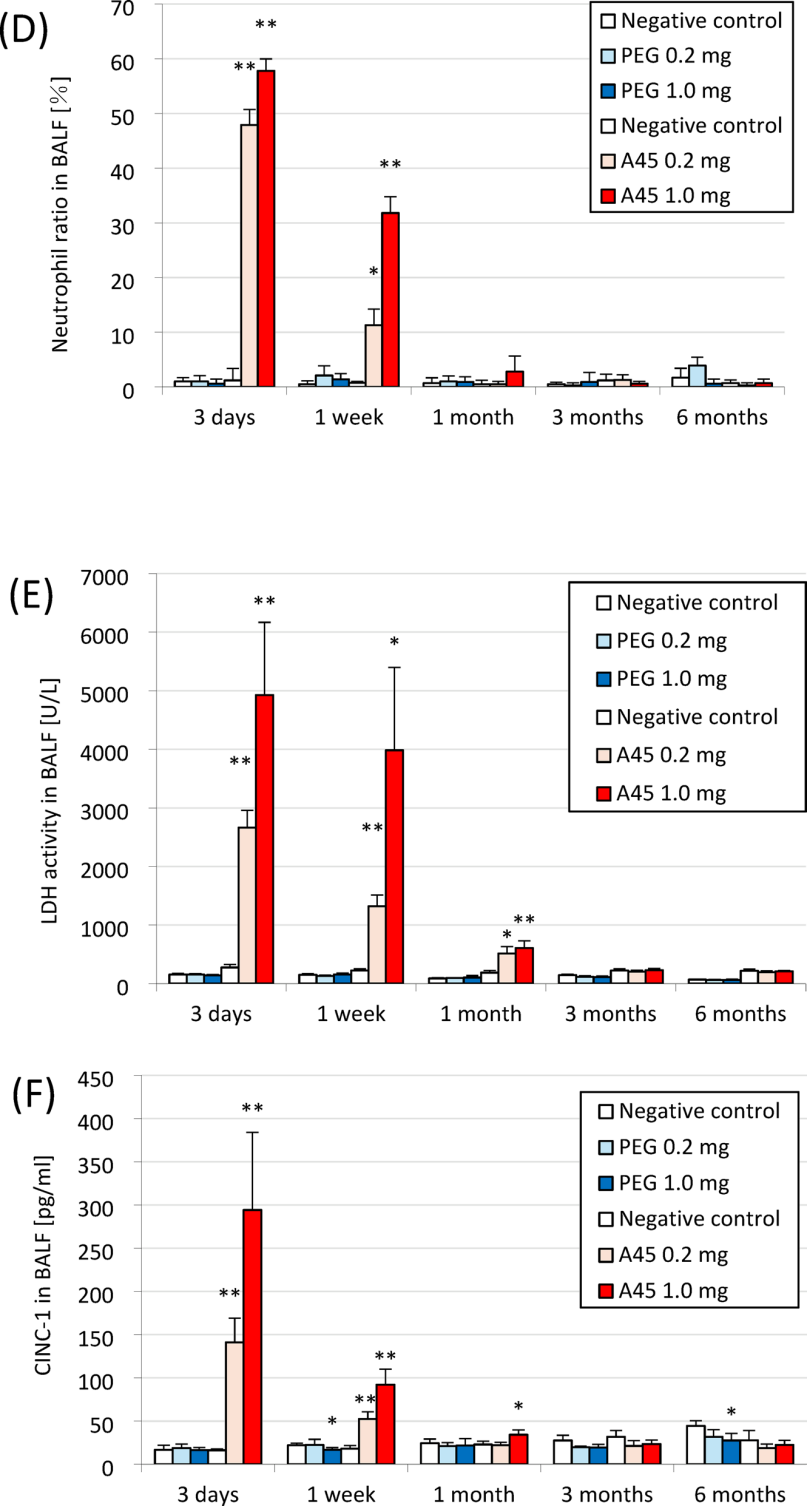

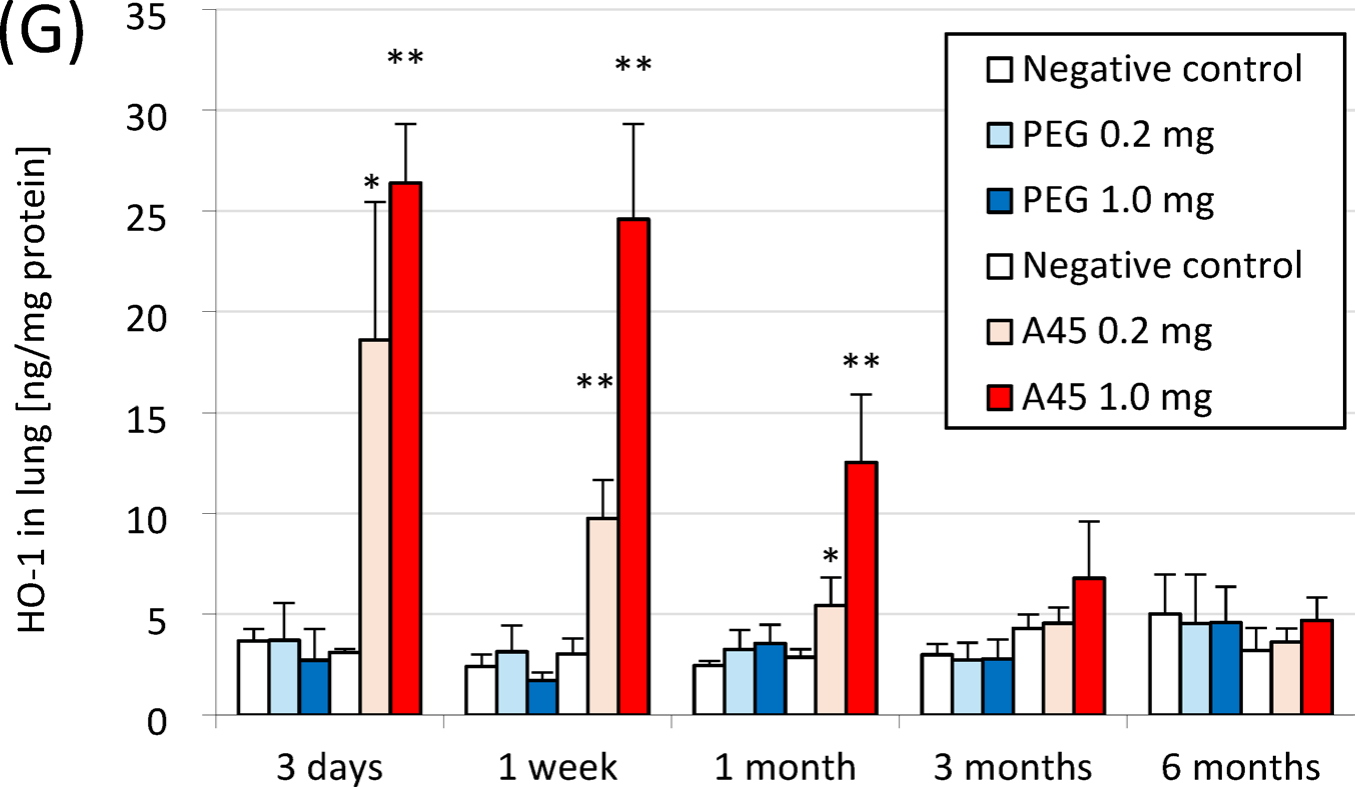
Results of intratracheal instillation studies of PEG and A45. (**A**) Body weight change ratio. (**B**) Total cell counts in BALF. (**C**) Neutrophil count in BALF. (**D**) Percentage of neutrophils in BALF. (**E**) LDH activity in BALF. (**F**) CINC-1 concentration in BALF. (**G**) HO-1 concentration in lung tissue. Data are mean ± SD for *n* = 5/group (* *p* < 0.05, ** *p* < 0.01).

The relative body weight (body weight at each observation time divided by the baseline value before instillation) was significantly decreased at 3 days and 1 week after high-dose A45 exposure (*p* < 0.05). The body weight subsequently increased and returned toward baseline from 1 month after exposure. In contrast, no significant changes in body weight were observed in the PEG-exposed groups (Fig. 1A).

### Cell counts and inflammatory markers in BALF

The results of cell analysis and inflammatory markers in PAA have been published before^22^. Significant increases (*p* < 0.05) in the total number of cells, neutrophils, and the proportion of neutrophils were observed in all the A45-exposed groups from 3 days to 1 month after exposure compared with their respective controls (Fig. 1B–D). In addition, the LDH activity and CINC-1 levels in the BALF, as well as the HO-1 levels in lung tissue, tended to increase during the same period (Fig. 1E–G). These results indicate that A45 induced lung inflammation and injury persisting for at least 1 month after exposure. By contrast, no significant changes were observed in the PEG-exposed groups compared with the negative controls.

### Histopathological findings in the lung

**Fig. 2:**
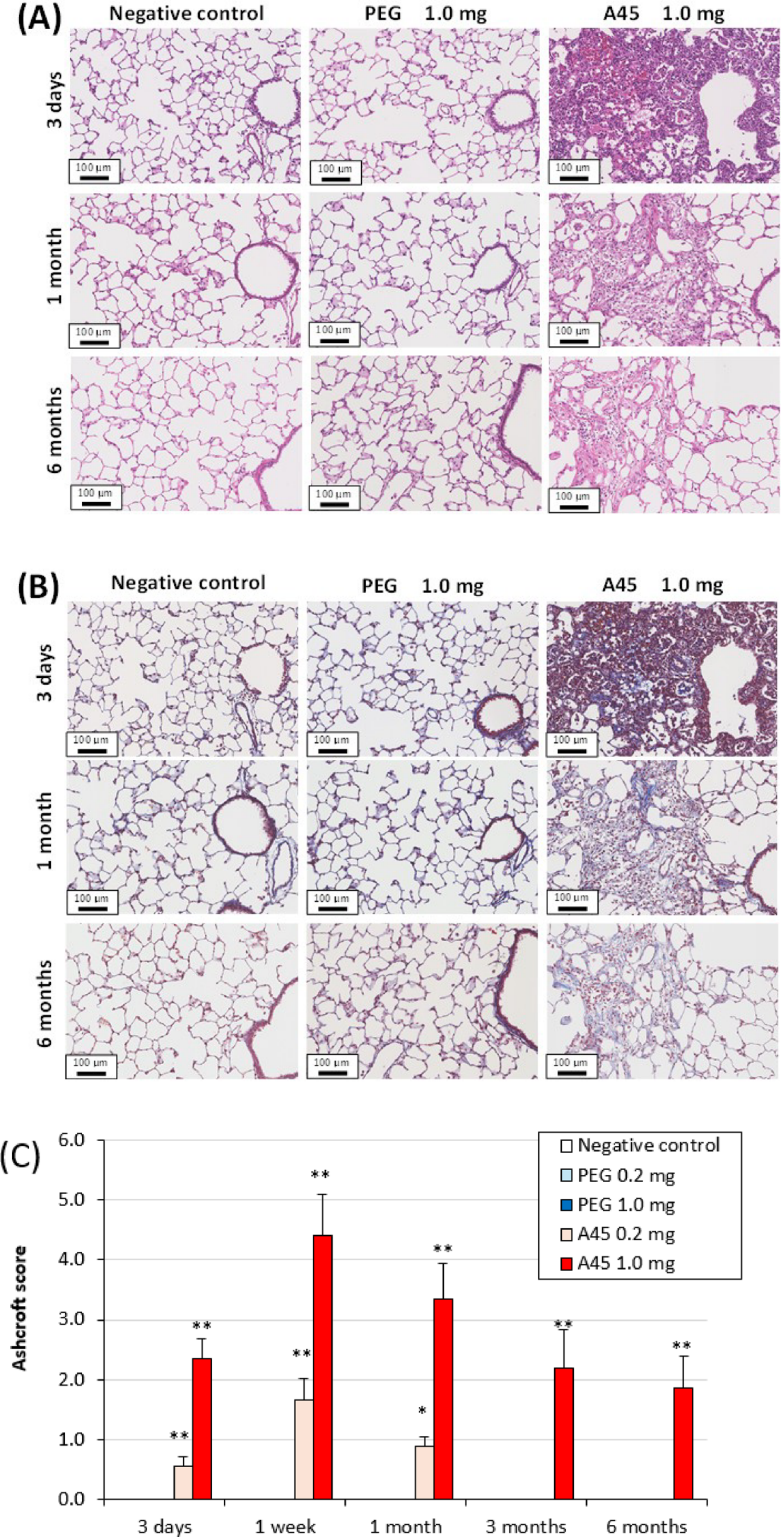
Histopathological findings in lungs exposed to PEG and A45. (**A**) Representative HE staining at 3 days, 1 month, and 6 months after instillation. (**B**) Representative MT staining at the same time points. (**C**) Ashcroft scores of lung fibrosis.

Representative histopathological findings at 3 days, 1 month, and 6 months after PEG or A45 instillation are shown in Fig. 2. Marked neutrophil infiltration into the alveoli was evident in a dose-dependent manner in the A45-exposed lungs at 3 days (Fig. 2A). Fibroinflammatory changes were observed from 3 days to 1 month after exposure, reflecting substantial lung injury. Although fibrosis gradually improved, it persisted for up to 6 months after exposure (Fig. 2B). In contrast, the PEG-exposed lungs showed no detectable inflammatory or fibrotic changes.

Quantitative evaluation using the modified Ashcroft score demonstrated a dose-dependent increase in fibrosis in the A45-exposed groups up to 6 months post-exposure (*p* < 0.05), whereas no increase was observed in the PEG groups throughout the observation period (Fig. 2C).

### Global metabolomic alterations

**Fig. 3:**
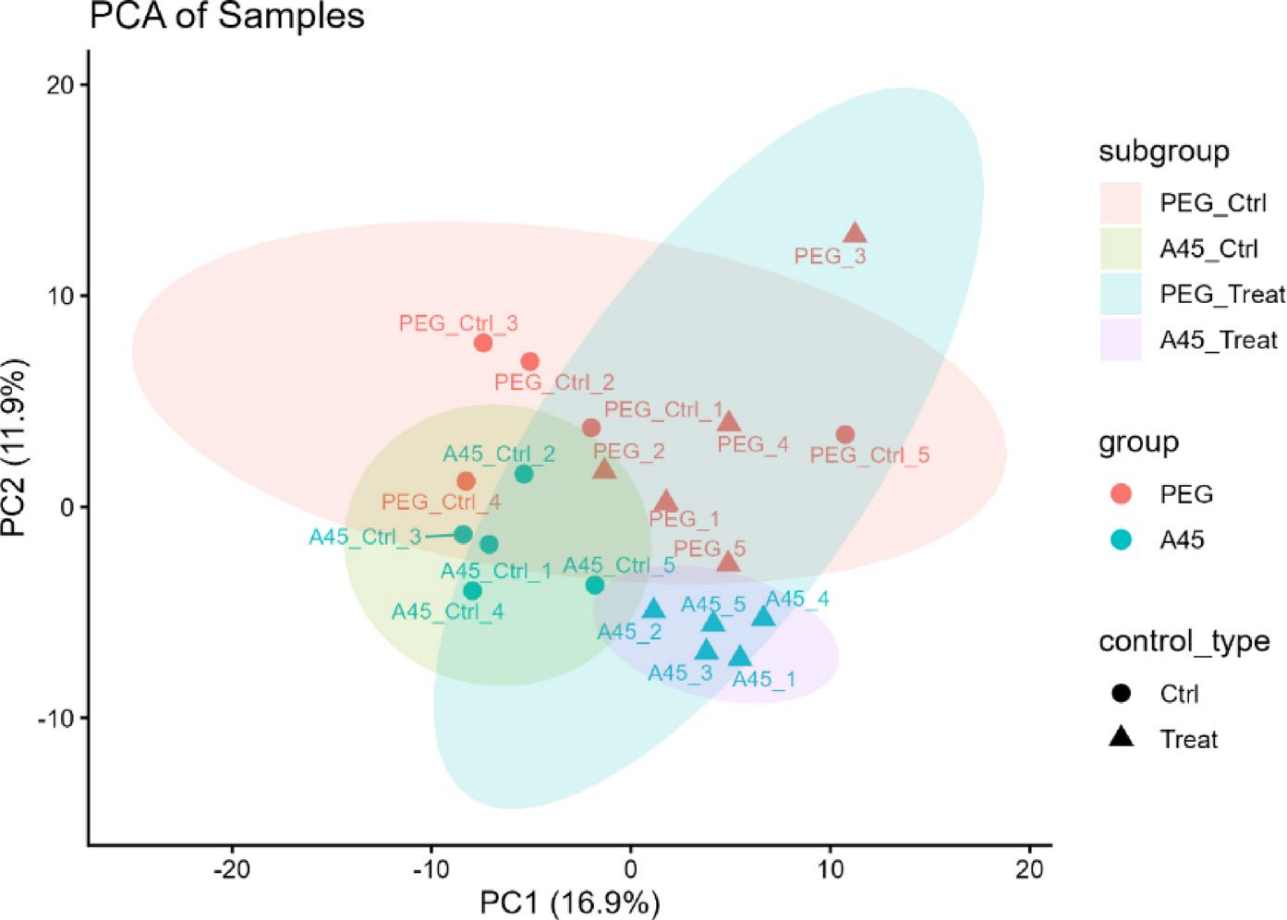
Principal component analysis of metabolomic profiles. Principal component analysis (PCA) score plot showing partial separation of A45-treated rats from their matched controls, whereas PEG-treated rats clustered closely with their corresponding controls. PC1 and PC2 explained 16.9% and 11.9% of variance, respectively.

A total of 249 metabolites were detected, of which 182 passed quality filtering. Principal component analysis (PCA) (Fig. 3A) revealed partial separation of A45-treated rats from their matched controls, whereas PEG-treated rats clustered closely with their corresponding controls. Because the two control groups (PEG Ctrl and A45 Ctrl) originated from independent experimental batches, we first compared them directly. PERMANOVA indicated no significant difference between the two control groups (*p* = 0.095, trend-level), and the dispersion test confirmed no difference in within-group variance.

To avoid potential batch-related confounding, all downstream analyses were conservatively performed with the control groups kept separate. This ensured that A45 treatment effects were evaluated relative to the A45 Ctrl group and PEG treatment effects relative to the PEG Ctrl group. The first and second principal components (PC1 and PC2) accounted for 16.9% and 11.9% of the total variance, respectively.

### Differential metabolites

**Fig. 4:**
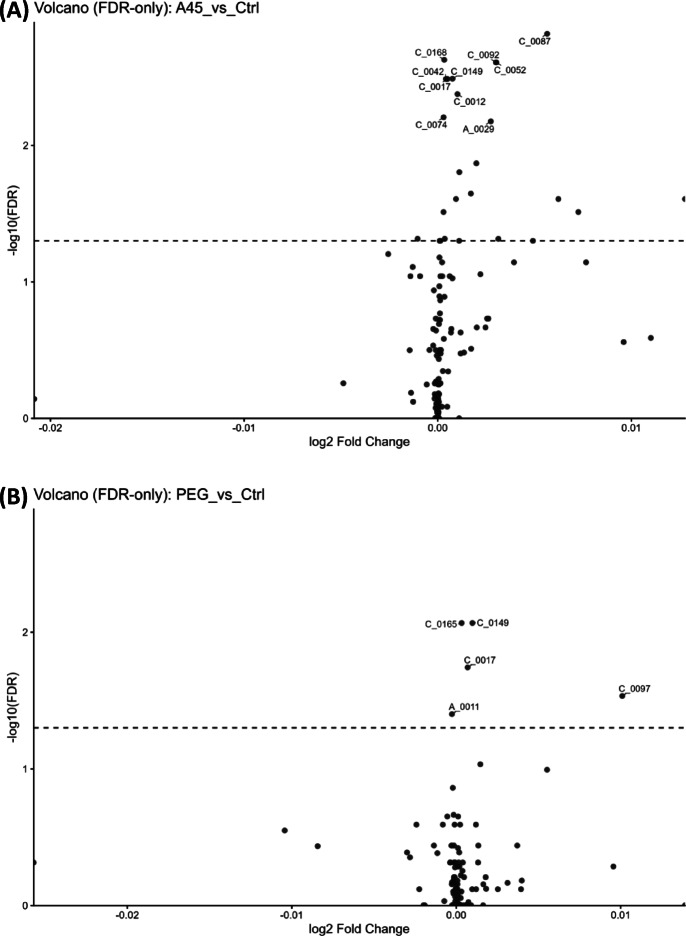

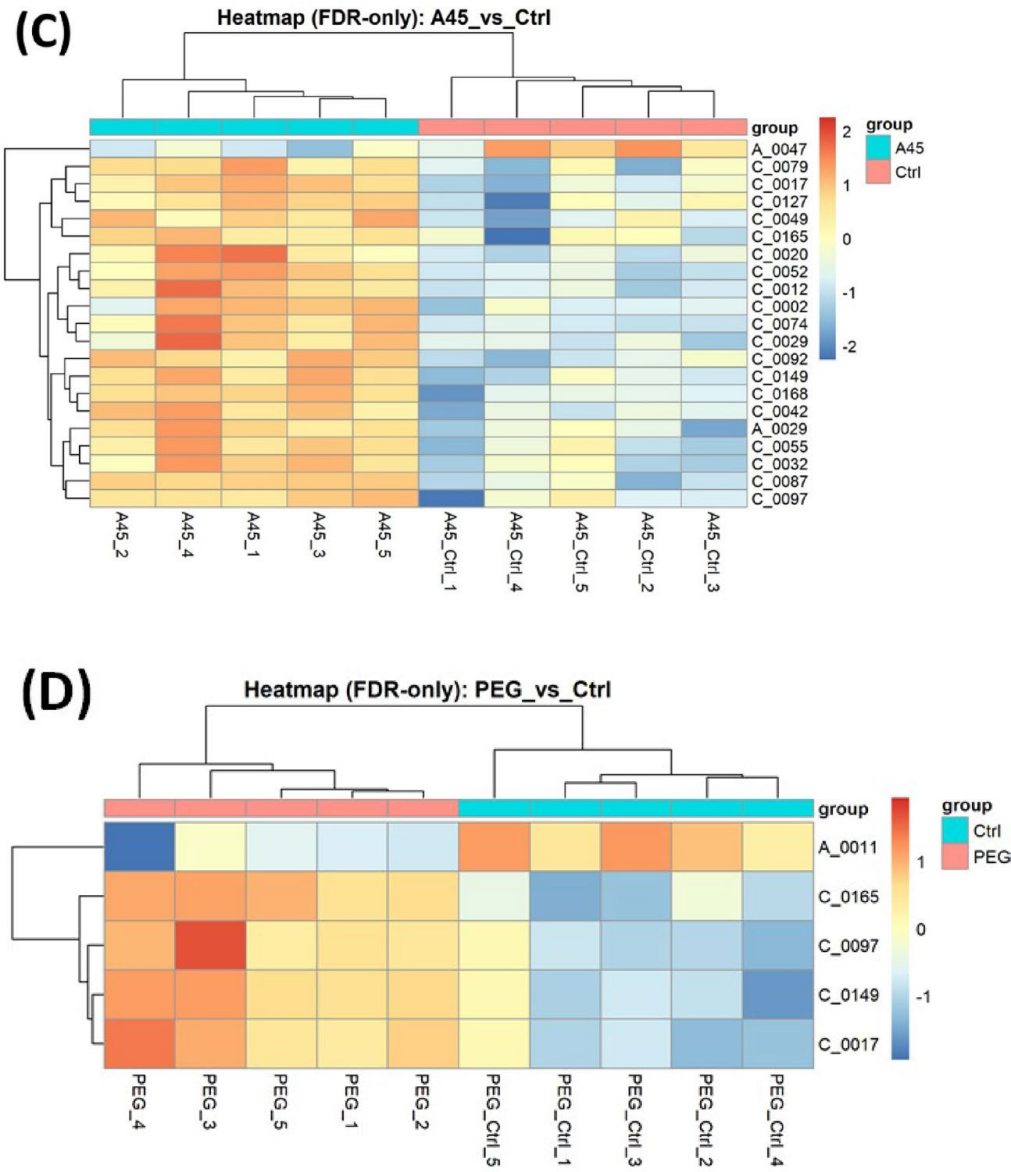
Differential metabolites. (**A**) Volcano plot for A45 versus A45 control. (**B**) Volcano plot for PEG versus PEG control.　Points represent individual metabolites; the x-axis shows log₂ fold change, and the y-axis shows − log₁₀(p-value). (**C**) Heatmap of differentially abundant metabolites between A45-treated rats and their matched controls. (**D**) Heatmap of PEG-treated rats and their controls. Multiple testing was controlled by the Benjamini–Hochberg procedure, with significance defined as FDR < 0.05. Labeled IDs correspond to metabolite identifiers used in this study; their correspondence with compound names is provided in Table 2 and Supplementary Table S1.

In the volcano analysis comparing A45-treated and A45 control groups, 21 metabolites passed the FDR < 0.05 threshold (Fig. 4A; Table 2). Similarly, in the PEG versus PEG control comparison, five metabolites were significant (Fig. 4B; Table 2). The heatmap of A45 versus A45 control revealed 21 metabolites with significant alterations (FDR < 0.05). These included amino acids and energy-related metabolites such as histidine, asparagine, alanine, proline, serine, valine, 2-aminobutyric acid, N-acetylleucine, malic acid, γ-butyrobetaine, and carnitine (Table S2). The clustering pattern showed a clear separation between A45-treated rats and their matched controls (Fig. 4C).

**Table 2:** Serum metabolites significantly altered by A45 or PEG exposure compared with respective controls (FDR < 0.05).

Comparison	Metabolite ID	Metabolite name	log_2_FC	FDR
A45_vs_Ctrl	C_0087	His	0.0056	0.0015
A45_vs_Ctrl	C_0168	N-(1-Deoxy-1-fructosyl)valine	0.0003	0.0024
A45_vs_Ctrl	C_0092	Carnitine	0.0030	0.0025
A45_vs_Ctrl	C_0052	Asn	0.0030	0.0025
A45_vs_Ctrl	C_0149	Cytidine	0.0008	0.0032
A45_vs_Ctrl	C_0042	3-Hydroxy-2-methyl-4-pyrone	0.0004	0.0033
A45_vs_Ctrl	C_0017	2-Aminoisobutyric acid 2-Aminobutyric acid	0.0005	0.0033
A45_vs_Ctrl	C_0012	Ala	0.0010	0.0042
A45_vs_Ctrl	C_0074	γ-Butyrobetaine	0.0003	0.0062
A45_vs_Ctrl	A_0029	Malic acid	0.0027	0.0067
A45_vs_Ctrl	C_0055	Leu	0.0020	0.0135
A45_vs_Ctrl	C_0032	Val	0.0011	0.0157
A45_vs_Ctrl	C_0049	Hydroxyproline	0.0017	0.0225
A45_vs_Ctrl	C_0029	Pro	0.0128	0.0247
A45_vs_Ctrl	C_0079	Gln	0.0009	0.0247
A45_vs_Ctrl	C_0020	Ser	0.0062	0.0247
A45_vs_Ctrl	C_0002	Ethanolamine	0.0003	0.0308
A45_vs_Ctrl	C_0097	Phe	0.0073	0.0308
A45_vs_Ctrl	A_0047	Uric acid	−0.0010	0.0484
A45_vs_Ctrl	C_0165	γ-Glu-Gln	0.0004	0.0484
A45_vs_Ctrl	C_0127	O-Acetylcarnitine	0.0031	0.0484
PEG_vs_Ctrl	C_0165	γ-Glu-Gln	0.0003	0.0086
PEG_vs_Ctrl	C_0149	Cytidine	0.0010	0.0086
PEG_vs_Ctrl	C_0017	2-Aminoisobutyric acid 2-Aminobutyric acid	0.0007	0.0181
PEG_vs_Ctrl	C_0097	Phe	0.0101	0.0293
PEG_vs_Ctrl	A_0011	Glyceric acid	−0.0003	0.0397

In contrast, the PEG versus PEG control comparison showed five significantly altered metabolites (Table S3), and the corresponding heatmap (Fig. 4D) demonstrated that PEG-treated rats remained intermixed with their controls, indicating a minimal metabolic impact of PEG treatment. Finally, a direct comparison between A45- and PEG-treated groups revealed four metabolites (C_0120, C_0047, C_0072, and A_0060) with differential abundance. The heatmap (Supplementary Figure S1) showed distinct clustering between the two treatment groups, further confirming that the observed metabolic alterations were specific to A45 exposure.

**Fig. 5:**
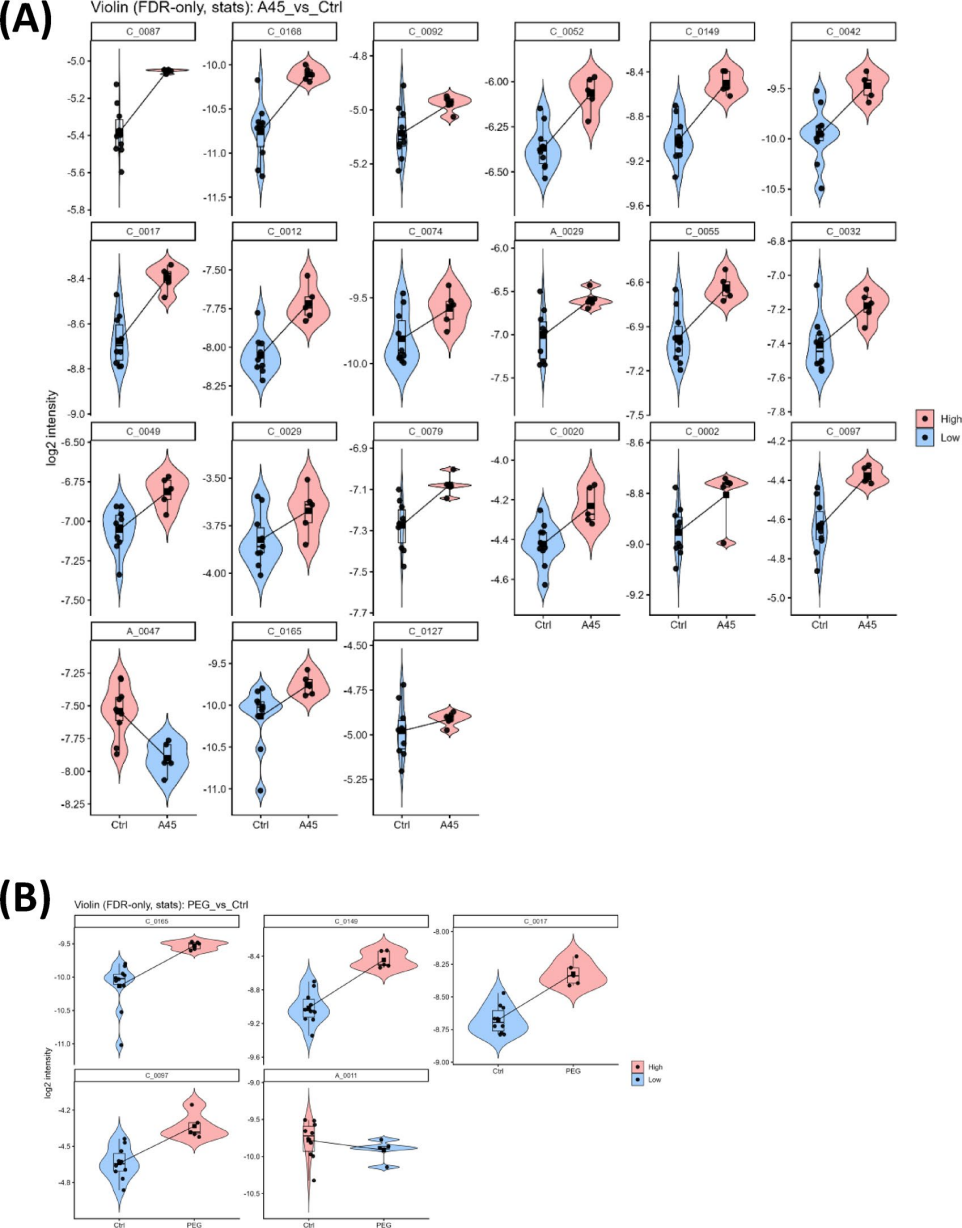
Boxplots and violin plots of Metabolites. Metabolites identified as significantly altered (FDR < 0.05) in (**A**) A45-treated rats relative to their matched controls and (**B**) PEG-treated rats relative to their matched controls are shown. Intensities were normalized across samples, with boxplots representing the median and interquartile range and violin plots showing the distribution within groups. Compound IDs shown in the panels correspond to metabolite names listed in Table 2.

To identify discriminant features specific to A45 exposure, violin plots demonstrated clear separation between A45-treated rats and controls across all 21 metabolites, with consistent upregulation or downregulation at the individual animal level (Fig. 5A). These results highlight a robust set of A45-specific biomarker candidates, reflecting perturbations in amino acid and energy metabolism as well as potential links to oxidative stress and inflammation. On the other hand, five metabolites were altered by PEG exposure, and four of these overlapped with those changed by A45 exposure (Fig. 5B). The consistent pattern across biological replicates supports the potential utility of these metabolites as candidate indicators of A45-induced metabolic perturbations.

### Pathway analysis visualized by Sankey diagrams

**Fig. 6:**
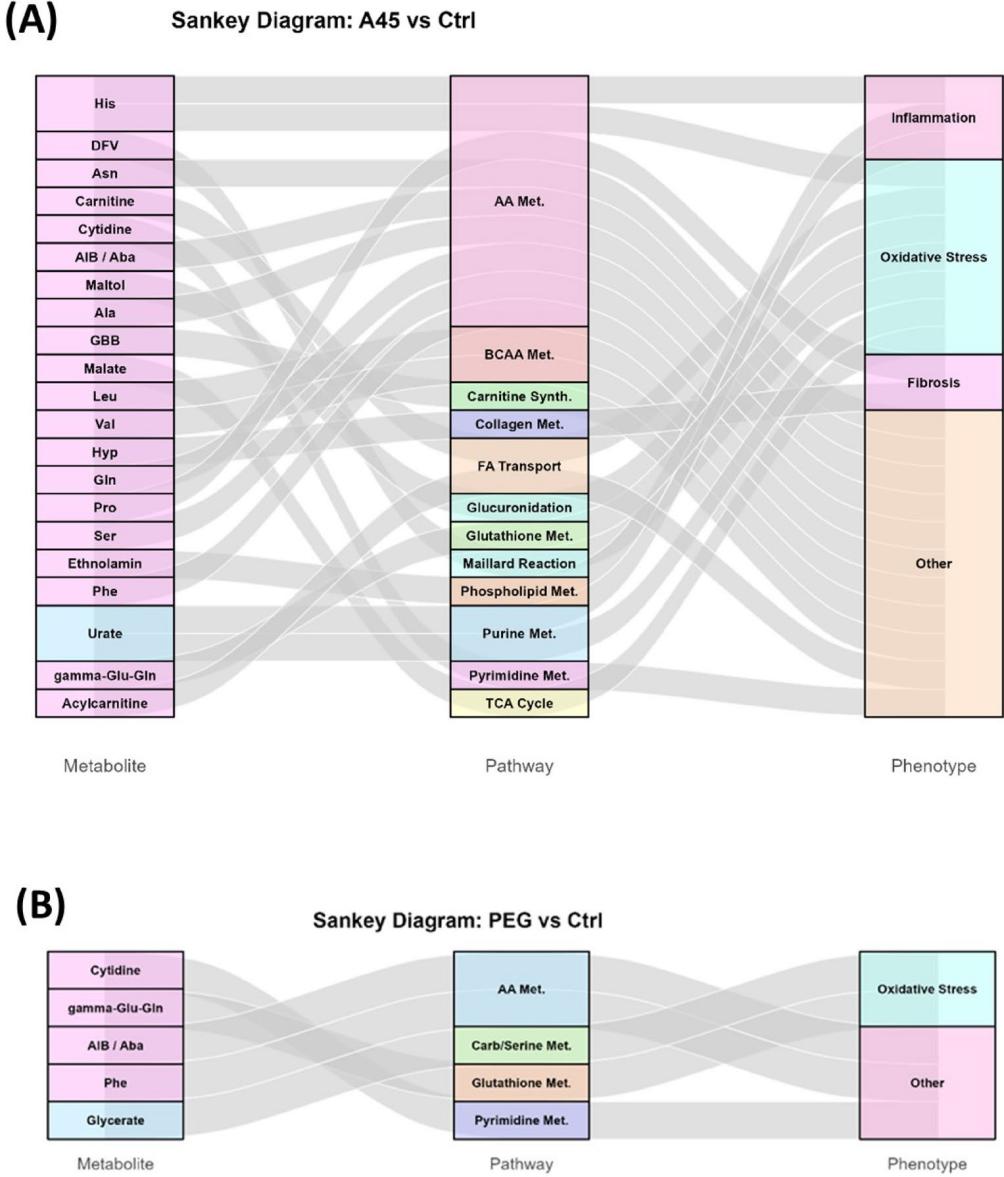
Sankey diagram representation of metabolite–pathway–phenotype relationships across two comparisons. (**A**) A45 vs. control (21 significantly altered metabolites; 20 upregulated, 1 downregulated). (**B**) PEG vs. control (5 significantly altered metabolites; 4 upregulated, 1 downregulated). Differential metabolites (FDR < 0.05) were mapped to KEGG pathways and grouped into higher-level phenotype categories. Link widths vary visually depending on layout, but do not encode quantitative information. Metabolites on the left axis are ordered by decreasing |log₂FC| (top = larger |log₂FC|), with red indicating upregulation and blue indicating downregulation.

Sankey diagrams were used to link significantly altered metabolites to KEGG pathways and higher-level phenotype categories (Fig. 6).

For the A45 vs. Ctrl comparison, the flows formed coherent streams from amino-acid and energy-metabolism nodes (e.g., branched-chain and urea-cycle–related metabolites, TCA intermediates, and the γ-glutamyl/glutathione axis) toward phenotypes related to oxidative stress and inflammation.

Several lipid- and membrane-associated nodes (e.g., ethanolamine/PE synthesis) also contributed to inflammatory phenotypes, consistent with membrane remodeling under redox stress (Fig. 6A).

Collectively, the diagram indicates that A45 exposure elicits pathway-level reprogramming that converges on oxidative stress and inflammation, with enrichment of processes often linked to fibrotic remodeling (e.g., amino-acid/collagen-related and energy-demanding pathways), supporting a plausible connection between A45 exposure and fibrosis-related biology.

In contrast, PEG exposure caused only sparse and scattered changes (Fig. 6B), a pattern consistent with a minimal impact of PEG treatment on overall pathway organization.

## Discussion

In this study, we investigated metabolomic alterations associated with polyacrylic acid (A45)–induced lung injury and fibrosis using CE-TOFMS profiling in a rat intratracheal instillation model.

The human exposure equivalent to the maximum dose (1 mg) used for intratracheal instillation in this study was estimated using a previously published formula^31^:

(amount of PAA) = (particle concentration) × (tidal volume) × (breathing frequency) × (daily exposure hours) × (deposition fraction).

Assuming a lung deposition rate of 0.1 for both humans and rats, exposure to the ACGIH TLV-TWA for general dust (3 mg/m³) would correspond to approximately 926 days of exposure, based on tidal volumes of 2.1 and 625 mL; breathing frequencies of 102 and 12 times/min; and daily exposure durations of 6 h (rat and human lung weights set at 1 g and 1000 g). Under typical work conditions (3 mg/m³ for 8 h/day, 5 days/week), a tracheal dose of 1 mg corresponds to about 3.6 years of exposure. In actual workers, lung disorders have been reported after more than two years of exposure to acrylic acid–based polymers^7^, suggesting that the exposure level used in this study is relevant to real-world conditions.

Our findings demonstrated that A45, but not polyethylene glycol (PEG), elicited significant metabolic perturbations, particularly in amino acid, energy, and redox-related pathways, as well as connections to inflammation- and fibrosis-related phenotypes, consistent with its high fibrogenic potential. 21 metabolites in A45 exposure group met the FDR threshold in the volcano plot analysis; these metabolites showed significant changes compared to the control in the heatmap analysis, highlighting robust and reproducible alterations. In contrast, PEG exposure resulted in only sparse and scattered changes, reinforcing its relative metabolic inertness. These results highlight the utility of metabolomics in elucidating mechanisms of chemically induced lung disorders and in distinguishing toxic from biocompatible polymers.

A methodological issue requiring consideration is the handling of control groups. In this study, the PEG control and A45 control rats originated from separate experimental batches. To address potential batch effects, we directly compared the two control groups using PCA and PERMANOVA. Although no significant differences were observed (*p* = 0.095, trend-level), we conservatively treated the PEG Ctrl and A45 Ctrl as independent reference groups in all downstream analyses. This strategy avoided confounding across batches and ensured that treatment effects were assessed relative to the appropriate matched control. Importantly, the lack of a significant difference between the control groups supports their comparability, but the explicit separation strengthens the robustness of our conclusions.

Consistent with our previous work^22^, polyacrylic acids caused severe lung inflammation within 3 days to 1 month after instillation, followed by resolution of the acute inflammatory phase and the onset of fibrotic remodeling. Although this study involved a single intratracheal instillation and fibrosis gradually improved during the chronic phase, this temporal pattern parallels observations in other models of acute lung injury, such as bleomycin- or LPS-induced injury, as well as in clinical cases of ARDS, where acute inflammation subsides but fibrotic pathways remain active and remodeling continues^32–34^. In contrast, the PEG-treated rats showed no significant lung inflammation or fibrosis up to 6 months, in agreement with reports that PEG exhibits low pulmonary toxicity and minimal activation of alveolar macrophages^35^. These divergent outcomes support the interpretation that polyacrylic acids possess a strong fibrogenic potential, whereas PEG serves as an appropriate negative control.

Metabolomic profiling provided additional mechanistic insights into these pathological changes. Amino acid metabolism emerged as the most prominently altered pathway following A45 exposure. In our dataset, leucine, alanine, and asparagine were significantly increased, indicating perturbations in protein turnover and energy metabolism. In line with previous studies, increases in proline and serine have been implicated in enhanced collagen synthesis consistent with TGF-β–driven fibrogenesis^14,36–38^, suggesting that similar metabolic reprogramming may underlie the fibrotic response to A45. Alterations in branched-chain amino acids (notably leucine and valine) further point to disrupted energy balance. Interestingly, increased levels of BCAAs at 1 month may also reflect recovery of food intake after initial weight loss, aligning with clinical observations where BCAAs fluctuate between depletion in acute illness and rebound during recovery^39–44^. These findings suggest that systemic amino acid metabolism dynamically reflects both injury and repair phases.

Energy metabolism was also significantly affected, with altered malate pointing to dysregulation of the tricarboxylic acid (TCA) cycle. Mitochondrial dysfunction has been implicated in ROS generation, impaired ATP production, and chronic inflammatory responses in fibrotic lung disease, and our data are consistent with these mechanisms^45^. Parallel changes in alanine metabolism were also detected, echoing reports from serum metabolomics in silicosis patients^15^, suggesting shared metabolic signatures between A45-induced lung injury and fibrotic pneumoconioses.

Oxidative stress–related pathways were evident through increases in cysteine, methionine, and methionine sulfoxide. These metabolites are closely linked to glutathione biosynthesis and antioxidant defense^46^, and their upregulation may represent compensatory responses to polyacrylic acid-induced oxidative stress. This interpretation is supported by elevated HO-1 levels and our previous observations of oxidative and ER stress induced by polyacrylic acid in alveolar macrophages^47,48^.

Fibrosis-related metabolic pathways may also be involved. Although lysine itself was not significantly altered in our dataset, we observed an increase in ethanolamine, implicating perturbation of glycerophospholipid metabolism and altered membrane lipid turnover^49,50^. This finding is consistent with lipidomic studies in idiopathic pulmonary fibrosis (IPF)^17,51^ and with serum metabolomics in silica- and asbestos-exposed patients^15^, suggesting that polyacrylic acid-induced fibrosis shares common metabolic hallmarks with occupational and idiopathic fibrotic lung diseases.

An important consideration is that local metabolic alterations in the lung are not always directly mirrored in the circulation. Many metabolites generated or consumed within lung tissue may undergo rapid turnover, degradation, or compartmentalization, limiting their detectability in serum. Nevertheless, several classes of metabolites—including amino acids, carnitine, and energy-related intermediates—are readily released into the bloodstream and have been reported as systemic indicators of lung fibrosis or injury in both experimental models and patients^13,52^. Moreover, systemic metabolic responses to lung inflammation, such as alterations in branched-chain amino acids or redox-related metabolites, can amplify disease signatures in the circulation^53,54^. Thus, the serum metabolomic profiles obtained in this study should be interpreted not as direct reflections of lung-tissue metabolism, but rather as integrated systemic readouts of pulmonary injury and fibrotic remodeling, which is also advantageous for identifying clinically accessible biomarkers.

Several limitations in this study should be acknowledged. First, the metabolomic analyses were performed at a single time point (1 month), precluding separation of early inflammatory versus later fibrotic signatures. Time-series metabolomics would strengthen mechanistic inference. Second, fibrosis was confirmed by histopathology but not by molecular markers such as TGF-β1 or collagen isoform expression. Integration of transcriptomic and proteomic approaches could provide a more comprehensive systems-level view. Third, although no statistically significant differences were detected between the two control groups, the fact that they were derived from different experimental batches necessitated their separate treatment in the analyses. This conservative approach may have reduced statistical power but increased robustness by avoiding batch-related confounding. Finally, in this study we analyzed metabolic alterations using serum samples, which were selected as a minimally invasive and clinically accessible matrix to capture systemic metabolic signatures of acrylic acid polymer exposure. However, serum metabolomics provides only indirect information on pulmonary injury. Because lung tissue and BALF were not evaluated in parallel, the relationship between local pulmonary metabolic changes and systemic metabolite profiles remains unclear. Future studies integrating both lung-specific (lung tissue and BALF) and circulating metabolites will be necessary to fully elucidate the metabolic mechanisms underlying acrylic acid polymer–induced lung injury.

In addition, although PEG is a well-known laboratory contaminant in MS-based analyses, several aspects of our protocol (PEG-free extraction reagents, 5-kDa ultrafiltration, and the use of a primary metabolite–focused CE-TOFMS platform) make PEG-related artifacts unlikely. The limited number of changes observed in the PEG vs. PEG control comparison further supports the conclusion that the serum metabolomic alterations reported here primarily reflect biological effects of A45 exposure rather than technical contamination.

Despite these limitations, our findings highlight the value of metabolomics for mechanistic toxicology and for identifying candidate biomarkers of polymer-induced lung injury.

## Conclusion

Our study demonstrates that A45, but not PEG, induces distinct metabolomic reprogramming in pathways related to amino acid, energy, and redox metabolism, which are consistent with　the transition from acute inflammation to fibrosis. By explicitly evaluating and separately analyzing the two control groups, we ensured the robustness of our conclusions and minimized potential batch confounding. Moreover, the identification of A45-specific biomarker candidates provides a foundation for future translational studies aimed at developing metabolic indicators of polymer-induced lung injury.

## Supplementary Information

Below is the link to the electronic supplementary material.

Supplementary Material 1

Supplementary Material 2

Supplementary Material 3

Supplementary Material 4

## Data Availability

The datasets used during and/or analyzed during the current study are available from the corresponding author on reasonable request.

## Declarations

### ARRIVE statement

The reporting of animal experiments is in accordance with the ARRIVE guidelines.
