# Supplementary material for: Polyacrylic acid, but not polyethylene glycol, induces metabolic reprogramming linked to pulmonary fibrosis in rats
Source: Sci Rep. 2025 Dec 18;16:3215. doi: 10.1038/s41598-025-33213-1 (PMC12830845; doi:10.1038/s41598-025-33213-1)
Supplement: Supplementary file 4 — Supplementary Material 4 [file 41598_2025_33213_MOESM4_ESM.pptx]

## Slide 1
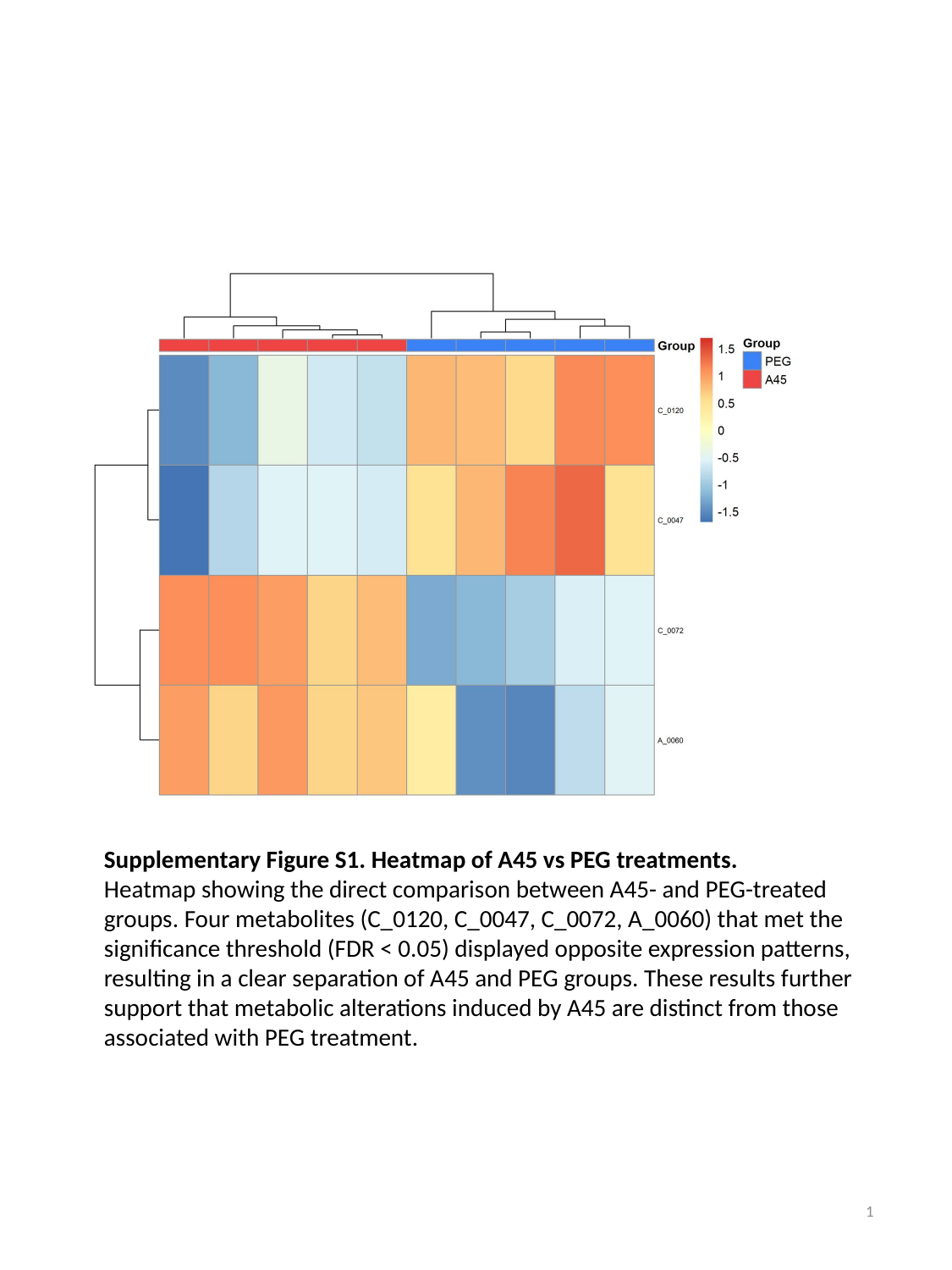

Supplementary Figure S1. Heatmap of A45 vs PEG treatments.
Heatmap showing the direct comparison between A45- and PEG-treated groups. Four metabolites (C_0120, C_0047, C_0072, A_0060) that met the significance threshold (FDR < 0.05) displayed opposite expression patterns, resulting in a clear separation of A45 and PEG groups. These results further support that metabolic alterations induced by A45 are distinct from those associated with PEG treatment.
1
